# Participation in physical activity and life skills in older adults: sequential roles of resilience and ego integrity

**DOI:** 10.3389/fpubh.2026.1878746

**Published:** 2026-08-04

**Authors:** Hyojung Kwon, Inwoo Kim

**Affiliations:** 1 Department of Life Physical Education, College of Health and Bio, Semyung University, Jecheon, Republic of Korea; 2 Department of Sports Science Convergence, Dongguk University, Seoul, Republic of Korea

**Keywords:** ego integrity, life skills, older adults, physical activity, resilience

## Abstract

**Background:**

As the global population ages, fostering adaptive capacities to resolve daily challenges has become a critical public health priority. While older adults’ participation in physical activity is known to promote healthy aging, the psychological processes through which it may be associated with practical life skills in older adults remain under-explored.

**Objective:**

This study investigated the sequential associations between participation in physical activity and life skills in older adults, focusing on the roles of resilience and ego integrity.

**Methods:**

A total of 279 older adults participated in this study. Older adults’ participation in physical activity was assessed based on the World Health Organization (WHO) threshold of 150 min of moderate-intensity activity per week. Resilience, ego integrity, and life skills in older adults were measured using validated scales. Data were analyzed using Hayes’ PROCESS Macro (Model 6) to test the hypothesized sequential association model.

**Results:**

The results indicated that physical activity was positively associated with resilience, which was in turn associated with both ego integrity and life skills in older adults. While the indirect path through ego integrity alone was non-significant, physical activity maintained a significant direct association with life skills. Most importantly, a statistically significant sequential indirect association was observed between physical activity and life skills through resilience and ego integrity.

**Conclusion:**

These findings suggest that resilience may represent an important psychological factor associated with the relationship between behavioral engagement and broader psychological development, ultimately being associated with the practical competencies required for successful aging. To maximize the enhancement of life skills in older adults, physical activity interventions should be intentionally designed to foster psychological resilience as a supportive factor for developmental processes in later life.

## 1 Introduction

With the extension of average life expectancy and the rapid increase in the older adult population, the focus of aging has shifted from “how long to live” to the qualitative aspect of “how to live well.” In most developed countries, life expectancy at birth is approximately 80 years, an increase of about 25 to 30 years since 1900 (1). Globally, the number of individuals aged 65 and older is projected to reach approximately 1.6 billion by 2050, accounting for 16.7% of the total population of 9.4 billion. This implies an annual increase of 27.1 million older adults between 2015 and 2050 (2). The phenomenon of population aging does not merely signify an increase in numbers; it has triggered various individual, economic, and social challenges, leading individuals, nations, and academia to focus on the quality of life in later years. If longevity is not accompanied by physical, psychological, and social health, the quality of life for older adults will inevitably decline. Consequently, there is significant interest across various disciplines in identifying factors that influence the quality of the aging process and finding methods to extend healthy life expectancy and delay aging. This reflects the paramount importance of the qualitative dimension of later life: not just living longer, but living healthily.

Historically, older adults were perceived as passive subjects, with research predominantly focusing on the deficit-based aspects of aging. Recently, however, a paradigm shift toward successful aging has emerged, suggesting that continuous development and self-growth remain possible throughout the lifespan (3). As part of this shift, scholarly attention has increasingly been drawn to the cumulative impact of life skills in older adults, which are viewed as a set of psychological and social capabilities essential for maintaining health and emotional well-being in later life (4). These skills encompass a multidimensional framework, including physical, psychological, and social competencies. Building upon the multidimensional model of life skills in older adults proposed by Kwon (5), which emphasizes the integration of these diverse domains, this study particularly focuses on physical activity as a fundamental element for enhancing such life skills. Within this framework, physical activity is identified as a cornerstone for navigating later life; maintaining functional fitness is not only a prerequisite for independent living but also a critical driver for enhancing overall life skills in older adults (6, 7). Recent evidence continues to underscore the cumulative benefits of physical engagement for psychological well-being in later life; for instance, Rejeski and Mihalko (8) emphasized that regular physical activity serves as a critical foundation for enhancing the quality of life and facilitating successful adaptation to the aging process.

To further clarify the concept and benefits of physical activity, this study defines physical activity as any bodily movement produced by the contraction and relaxation of skeletal muscles that results in energy expenditure (9). It refers to physical activities that individuals perform systematically and repeatedly with the goal of promoting health. Furthermore, older adults’ participation in physical activity was operationally defined as the volume of physical activity, calculated by multiplying the average weekly duration and frequency of moderate-to-vigorous physical activity.

Existing large-scale cohort studies and meta-analyses have demonstrated that engaging in at least approximately 150 min of moderate-intensity physical activity per week is significantly associated with reduced mortality and the prevention of chronic diseases (10, 11). In particular, threshold effects indicating significant reductions in mortality risk at 150–300 min of activity per week have been reported, and these findings have been consistently replicated in recent cohort and meta-analytic studies (12–14). These findings suggest that the relationship between physical activity and health outcomes may not be purely linear, but instead may exhibit nonlinear characteristics in which health benefits become more pronounced beyond a certain level of activity.

Based on this evidence, the World Health Organization (15) and other major public health organizations recommend that older adults engage in at least 150 min of moderate-intensity physical activity per week. This guideline has become one of the most widely adopted standards for physical activity in both public health and clinical contexts. Meeting or exceeding this threshold is associated not only with fundamental health benefits such as improved cardiovascular health, prevention of chronic diseases, and maintenance of musculoskeletal function but also with additional benefits as the amount of physical activity increases. Specifically, higher levels of physical activity are associated with reduced risks of cardiovascular and metabolic diseases, cancer, and mortality (15). In addition, physical activity is associated with improved mental health, including lower symptoms of depression, anxiety, and psychological distress (16–18), and may contribute to better late-life cognitive function, although recent meta-analytic evidence suggests that this association is modest (19). It also plays a crucial role in supporting functional ability and independent living among older adults (6). Overall, these findings suggest that participation in physical activity beyond a certain threshold may be associated not only with physical health benefits but also with psychological, cognitive, and functional advantages.

The focus on physical activity is further justified by the premise that participation in such activities transcends mere health promotion; it may support the acquisition of skills that are transferable to daily life, ultimately contributing to human flourishing (20, 21). Continuous engagement in physical activity may contribute to the development of essential life skills, where the values and competencies internalized during exercise transition into broader life contexts, enhancing overall quality of life (22). While the development of life skills through sport has been extensively investigated within the framework of Positive Youth Development (PYD) (23), research specifically targeting older adults remains disproportionately scarce. Recent empirical evidence has begun to address this gap. Kwon et al. (24) found that older adults’ participation in physical activity was positively associated with life skills in older adults and that life skills mediated the relationship between participation in physical activity and successful aging. This finding provides preliminary empirical support for the relevance of life skills in later life and suggests that physical activity may be an important behavioral factor associated with the development of life skills in older adults. Nevertheless, the psychosocial processes through which participation in physical activity is associated with life skills in older adults remain largely unexplored. The competencies required for successful adaptation differ across the lifespan. Older adulthood is characterized by unique developmental tasks and life challenges that differ substantially from those of earlier developmental stages. Therefore, from a lifespan developmental perspective, an age-specific conceptualization of life skills is required to adequately capture the adaptive competencies necessary for successful aging. In response to this need, Kwon (5) established the conceptual framework and measurement scale for life skills in older adults through literature review, qualitative inquiry with community-dwelling older adults, open-ended surveys, expert content validation, and confirmatory factor analysis. Based on this conceptual framework, life skills in older adults are defined as multidimensional adaptive competencies encompassing the attitudes and skills that enable older adults to effectively respond to age-related developmental tasks and everyday challenges while maintaining functional independence, psychological well-being, and active social participation (5). Specifically, this construct comprises five interrelated domains: physical activity performance skills (physical capability ranging from basic daily activities to vigorous physical activity), daily self-efficacy skills (confidence in performing daily and independent activities), adaptation and acceptance skills (flexible adaptation to physical and environmental changes), participation skills in social activities (engagement in socially interactive activities), and engagement skills in digital society (adaptation to a digital environment through the use of digital technologies). In support of this broader perspective, emerging systematic evidence provides foundational insights: sport participation among older adults has been synthesized to yield diverse psychological and social benefits, including enhanced self-confidence, self-esteem, and resistance to negative perceptions of aging (25). Furthermore, qualitative explorations of lifelong sports participation suggest that physical engagement fosters enduring competencies for self-management and social contribution (26). These findings collectively suggest that physical activity may serve as an important factor associated with the development of life skills in later life.

To clarify the conceptual relationship between resilience and life skills, resilience is positioned as an adaptive psychological resource that may be associated with the development and application of life skills in older adults. Life skills in older adults refer to multidimensional competencies that enable individuals to adapt to rapidly changing physical, psychological, social, and environmental conditions in later life. For these competencies to be effectively applied in daily life, older adults may need psychological resources that help them regulate emotions and behavior, flexibly respond to adversity, and recover from age-related challenges. Resilience is therefore considered a particularly relevant psychological resource in the present model. This positioning is consistent with contemporary resilience theory. Resilience is not conceptualized as a fixed personal trait, but as a dynamic process through which individuals negotiate, adapt to, and manage adversity by mobilizing personal, social, and environmental resources (27, 28). In gerontological research, Wister et al. (29) proposed a lifecourse model of multimorbidity resilience, emphasizing that older adults’ adaptation to health-related and age-related adversity involves the activation of resources accumulated across the life course. Similarly, a systematic review and correlational meta-analysis of community-living older adults showed that resilience is associated with a broad range of psychological, social, health-related, and environmental factors, supporting its multidimensional and contextual nature (30).

Resilience may be especially relevant in later life because older adults often face multiple forms of loss and transition, including changes in physical function, social roles, independence, and daily routines. At the same time, accumulated life experiences may provide older adults with adaptive resources such as emotional regulation, problem-solving ability, persistence, and positive reinterpretation (31). Empirical evidence suggests that older adults can show relatively high levels of resilience, particularly in emotional regulation and problem-solving, compared with younger adults (32). Reviews of resilience in later life also indicate that resilience is associated with successful aging, lower depression, longevity, and better adaptation to the adversities of aging (33). Within this theoretical framework, participation in physical activity is conceptualized as a behavioral context that may be associated with resilience-related resources. Physical activity may provide repeated opportunities for manageable challenge, self-regulation, persistence, bodily confidence, and social engagement (34, 35). A systematic review of physical activity and psychological resilience in older adults reported that physical activity is positively associated with resilience in later life (36). Therefore, the present study examines a theoretically guided associational pathway in which physical activity is associated with resilience, resilience is associated with ego integrity, and these psychological resources are associated with multidimensional life skills in older adults.

Importantly, the proposed model does not test reciprocal relationships between resilience and life skills, nor does it establish causal or temporal ordering. Physical, psychological, and social factors in later life are likely to interact dynamically and reciprocally over time. Accordingly, the sequence proposed in this study should be understood as a simplified analytical framework for examining one plausible pattern of statistical associations among physical activity, resilience, ego integrity, and life skills in older adults.

To further elucidate the relationship between physical activity and life skills, it is necessary to examine other influential psychological variables specific to later life. This study considers ego integrity as a critical psychological construct that uniquely characterizes the developmental tasks of old age. Ego integrity transcends transient life satisfaction; it encompasses a holistic acceptance of one’s past life and a reconciled perspective on mortality (37), representing the pinnacle of psychological maturation in late adulthood (38). According to Erikson’s theory, achieving this sense of wholeness promotes positive psychological development and adaptive functioning. Ego integrity is presumed to be intrinsically linked to life skills in older adults, particularly the “life acceptance” dimension, which involves maintaining a proactive lifestyle and an affirmative embrace of one’s current circumstances (5). Although direct empirical investigations linking these two constructs remain scarce, their shared emphasis on “acceptance” and “adaptive coping” provides a theoretical foundation for the proposed model. Furthermore, recent evidence suggests that higher ego integrity is associated with better functional adaptation and psychological resilience in the face of aging-related challenges (39–41).

From a lifespan developmental perspective, resilience is considered an important adaptive psychological resource in later life. Resilience enables older adults to adapt to age-related adversities, regulate negative emotions, and reconstruct meaning from challenging life experiences (27, 28). These adaptive psychological processes are theoretically relevant to ego integrity, which involves accepting one’s life as meaningful and coherent despite the inevitable losses and limitations associated with aging (37, 41). Accordingly, resilience may be associated with the developmental task of ego integrity by helping older adults integrate both positive and negative life experiences into a coherent life narrative. This perspective is consistent with positive psychology, which conceptualizes resilience as a dynamic capacity for positive adaptation and psychological growth across the lifespan (27). Therefore, resilience is expected to be positively associated with ego integrity. The positive cognitive appraisal, pursuit of meaning, and self-acceptance inherent in resilience may be relevant to developmental tasks appropriate for later life stages (41). Previous research suggests that resilience and ego integrity are positively related among older adults, indicating that adaptive psychological resources may support the integration of life experiences into a coherent and meaningful life narrative (39, 41–43). Consequently, this study hypothesizes that older adults’ participation in physical activity may be associated with life skills in older adults through a sequential pattern involving resilience and ego integrity. However, given the cross-sectional design, this sequence should be interpreted as a theoretically guided associational pathway rather than evidence of temporal or causal ordering.

**Figure 1:**
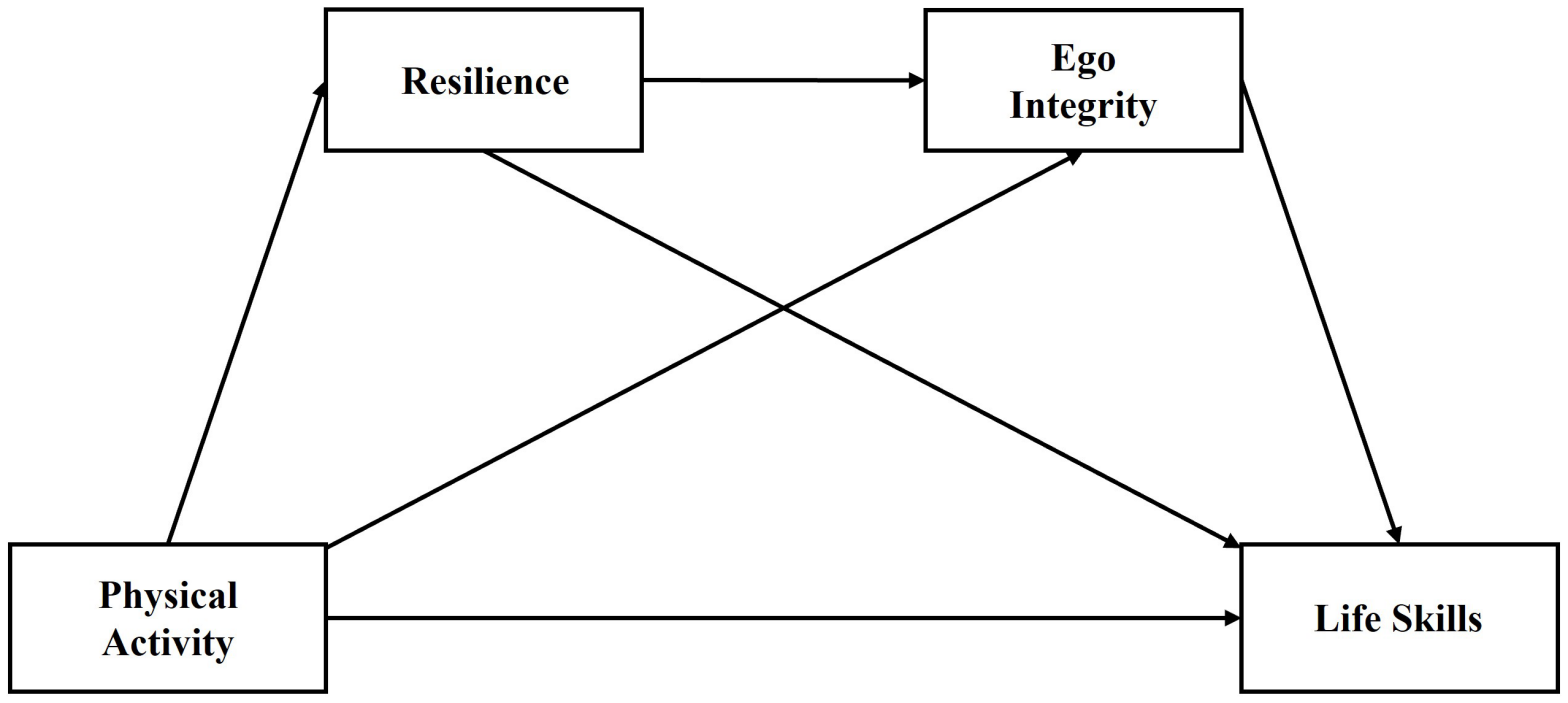
Conceptual model. The arrows represent theoretically guided statistical associations rather than causal or temporal effects.

Despite the clinical and social significance of these constructs, the framework for life skills in older adults has only recently been conceptualized, and integrated research examining the structural relationships among physical activity, resilience, ego integrity, and life skills in older adults remains limited. In particular, previous studies have largely examined these variables in isolation, with insufficient attention given to their potential sequential and interdependent functioning within a unified model. Therefore, the present study aims to extend the existing literature by examining a sequential model linking participation in physical activity to life skills in older adults through resilience and ego integrity. By integrating these variables within a single analytical framework, this study seeks to provide a more comprehensive understanding of the psychological mechanisms underlying successful aging. Specifically, this research contributes to the literature by (a) expanding the concept of life skills beyond youth populations to older adults, (b) empirically examining the sequential roles of resilience and ego integrity, and (c) offering a theoretically grounded explanation of how behavioral engagement may be associated with broader psychological development in later life. To further improve conceptual clarity, Figure 1 presents the theoretical model guiding the present study. In this model, participation in physical activity is positioned as a behavioral factor associated with psychological resources, resilience and ego integrity are positioned as intermediate psychological constructs, and life skills in older adults are positioned as multidimensional adaptive competencies reflecting physical, psychological, social, and digital participation-related capacities. The arrows in the figure indicate theoretically guided statistical associations rather than causal or temporal effects. This simplified sequence was chosen to test one plausible associational pathway grounded in resilience theory and lifespan developmental theory, while acknowledging that physical, psychological, and social factors in later life are likely to interact dynamically and reciprocally over time.

### 1.1 Research hypotheses

In light of the theoretical underpinnings discussed, the following hypotheses are proposed:

*H1*: Participation in physical activity will be positively associated with life skills in older adults.

*H2*: Resilience will be associated with the relationship between physical activity and life skills in older adults.

*H3*: Ego integrity will be associated with the relationship between physical activity and life skills in older adults.

*H4*: Resilience and ego integrity will be sequentially associated with the relationship between physical activity and life skills in older adults.

## 2 Methods

### 2.1 Participants and data collection

The participants of this study were older adults aged 65 and over, recruited from senior universities and community welfare centers in Seoul and Gyeonggi Province, South Korea. A purposive sampling method was employed to select individuals who had no cognitive impairments and were capable of effective communication. Prior to data collection, this study received ethical approval from the Institutional Review Board (IRB) of Dongguk University (Approval No. DUIRB2025-12-09).

To ensure cooperation, formal requests and study protocols were emailed to the administrators of relevant institutions. Data collection was conducted by the lead researcher and two trained research assistants, who provided a brief overview of the study’s purpose and obtained written informed consent from all participants. For participants who experienced difficulties in self-administration due to visual or physical constraints, the researchers read the questions aloud and recorded their responses as dictated, ensuring objectivity throughout the process. To minimize interviewer bias, the researchers were instructed to read each questionnaire item verbatim without providing additional explanations, interpretations, or suggestions and to record participants’ responses exactly as stated.

A total of 300 questionnaires were collected. After excluding responses that were incomplete or exhibited inconsistent response patterns, 279 samples were retained for the final analysis. The demographic characteristics of the final sample are presented in Table 1. This sample size met the minimum requirement calculated using G*Power 3.1 software for a regression-based mediation analysis with a medium effect size.

**Table 1:** Demographic characteristics of the participants (*N* = 279).

Characteristic	Category	*n*	%
Gender	Female	141	50.5
	Male	138	49.5
Age (years)	65–69	66	23.7
	70–74	44	15.8
	75–79	62	22.2
	80–84	64	22.9
	≥85	43	15.4
Perceived physical health	Very poor	9	3.2
	Poor	30	10.8
	Fair	139	49.8
	Good	91	32.6
	Very good	10	3.6
Perceived psychological health	Very poor	6	2.2
	Poor	24	8.6
	Fair	114	40.9
	Good	118	42.3
	Very good	17	6.1
Perceived economic status	Very poor	5	1.8
	Poor	21	7.5
	Fair	168	60.2
	Good	77	27.6
	Very good	8	2.9
Physical activity level	<150 min/week	119	42.7
	≥150 min/week	160	57.3

### 2.2 Measurements

#### 2.2.1 Participation in physical activity

To examine whether meeting international physical activity recommendations was associated with life skills in older adults, physical activity (PA) was assessed based on the World Health Organization guidelines (WHO, 2020). Participants reported their average weekly frequency of moderate-to-vigorous physical activity and the average duration per session using defined categorical intervals to minimize recall bias and ensure measurement reliability.

In the present study, physical activity referred to usual, self-selected health-promoting activities rather than participation in a standardized intervention program. The activities broadly included systematic and repeated moderate-to-vigorous activities performed for health promotion, such as walking, running, hiking, Tai Chi, swimming, other individually performed exercises, and organized exercise or sports programs. Thus, the physical activity variable was intended to capture the overall weekly volume of health-promoting physical activity rather than the effects of a specific exercise modality. The present study did not separately classify activity type, activity setting, or contextual and social characteristics, such as whether the activity was performed individually or in a group. Therefore, the observed associations should be interpreted as associations with overall physical activity participation based on weekly volume, not as effects of a particular type of physical activity or social participation context. Following the WHO criteria, which emphasize at least 150 min of moderate-intensity physical activity per week for older adults, the responses were transformed using midpoint values to determine the total weekly volume. This approach was intentionally designed to categorize participants into two distinct groups based on the 150-min threshold: those who met the recommended PA levels and those who did not. Accordingly, this variable was entered into the analytical model as a dummy-coded predictor (0 = <150 min; 1 = ≥150 min) to examine group differences in psychological outcomes between the two groups. Although physical activity participation was initially quantified using participants’ weekly frequency and duration of moderate-to-vigorous physical activity, the primary objective of this study was not to examine a dose–response relationship between physical activity volume and the outcome variables. Rather, the study aimed to compare older adults who met the internationally recognized WHO recommendation with those who did not. Therefore, the threshold-based categorization was theory-driven and based on an internationally established public health guideline rather than an arbitrary statistical criterion, thereby enhancing the practical interpretation and public health relevance of the findings.

#### 2.2.2 Resilience

Resilience was measured using the scale developed by Shin et al. (44). The 27-item instrument consists of nine sub-factors categorized into three primary domains: Control (cause analysis, emotional control, and impulse control), Positivity (gratitude, life satisfaction, and optimism), and Sociality (relationships, communication, and empathy). All items were rated on a 5-point Likert scale ranging from 1 (Strongly disagree) to 5 (Strongly agree). In the present study, the Cronbach’s alpha for the total scale was 0.909.

#### 2.2.3 Ego integrity

Ego integrity was assessed using the Ego-Integrity Scale originally developed by Kim (45) and further validated by Park (46). This 14-item scale measures four dimensions: satisfaction with current life, meaning and value of past life, acceptance of aging and death, and perceived utility of wisdom and experience. Each item was rated on a 5-point Likert scale. The Cronbach’s alpha for this scale was 0.910.

#### 2.2.4 Life skills in older adults

Life skills were evaluated using the Elderly’s Life Skills Scale (ELSS) developed by Kwon (5) and validated by Kwon et al. (24). The 21-item scale encompasses five factors: physical activity performance skills, daily self-efficacy skills, adaptation and acceptance skills, participation skills in social activities, and engagement skills in digital society. Responses were recorded on a 5-point Likert scale. The Cronbach’s alpha was 0.929, demonstrating high internal consistency.

### 2.3 Statistical analysis

Data analysis was performed using IBM SPSS Statistics 26.0 and the PROCESS Macro (Model 6) developed by Hayes (47). The specific analytical procedures were as follows:

First, descriptive statistics (mean, standard deviation, skewness, and kurtosis) were conducted to identify demographic characteristics and verify the normality of the data. Second, Cronbach’s alpha coefficients were calculated to ensure the internal consistency of the multi-item scales. Third, Pearson correlation analysis was performed to examine the relationships between the measured variables. Fourth, to evaluate potential multicollinearity, we examined the Tolerance and Variance Inflation Factor (VIF) values, ensuring they met the established psychometric criteria (Tolerance > 0.10; VIF < 10). Fifth, to assess the potential influence of common method bias, Harman’s single-factor test was conducted using an unrotated principal component analysis of the multi-item self-report scale items. Sixth, to examine the hypothesized model, we conducted an analysis using PROCESS Macro Model 6. This analysis aimed to explore whether meeting the WHO (15) physical activity threshold (150 min/week) was associated with life skills in older adults through a sequential pattern involving resilience and ego integrity. By utilizing the dummy-coded physical activity variable as the antecedent, we compared the differences in psychological outcomes between the groups that met versus did not meet the recommended guidelines. The statistical significance of the indirect associations was evaluated using bias-corrected bootstrapping with 5,000 resamples. An indirect association was considered statistically significant if the 95% confidence interval (CI) did not include zero.

## 3 Results

### 3.1 Descriptive statistics and correlation analysis

Descriptive statistics and correlation analysis were conducted to examine the general characteristics and interrelationships of the key variables. The results are summarized in Table 2. First, the normality of the data was assessed by examining the mean, standard deviation, skewness, and kurtosis of each variable. The skewness values ranged from −0.363 to 0.062, and the kurtosis values ranged from −0.171 to 0.503. Based on the criteria suggested by Kline (48), where absolute values of skewness <2 and kurtosis <7 indicate a normal distribution, the data in this study satisfied the assumption of univariate normality. To examine the potential presence of common method bias, Harman’s single-factor test was conducted. The unrotated principal component analysis showed that the first component accounted for 32.77% of the total variance, which was below the commonly used 50% threshold. This result suggests that common method bias was unlikely to be a serious concern in the present study.

**Table 2:** Descriptive statistics and correlation.

Variable		*M (SD)*	Skew.	Kurt.	Correlations			
					1	2	3	4
1. Physical activity (Dummy)		0.57 (0.50)	–	–	1			
2. Resilience		3.59 (0.45)	0.062	0.489	0.229**	1		
3. Ego integrity		3.71 (0.57)	−0.363	0.503	0.231**	0.738**	1	
4. Life skills		3.49 (0.61)	−0.099	−0.171	0.373**	0.716**	0.624**	1

*N* = 279. PA was dummy-coded (0 = <150 min, 1 = ≥150 min). **p < 0.01.

Next, Pearson correlation analysis was performed to identify the relationships among physical activity (PA) participation, resilience, ego integrity, and life skills in older adults. The results indicated significant positive correlations among all variables. Specifically, the correlation coefficients (*r*) ranged from 0.229 to 0.738 (*p* < 0.01). Since all correlation coefficients were below the threshold of 0.80, the data were deemed free from significant multicollinearity issues, ensuring the suitability of the variables for subsequent mediation analysis (48).

### 3.2 Regression-based mediation analysis

Prior to testing the hypothesized model, multicollinearity was assessed using Tolerance and the Variance Inflation Factor (VIF). All Tolerance values exceeded 0.452 and VIF values were below 2.214, confirming that multicollinearity did not bias the regression estimates (49).

**Figure 2:**
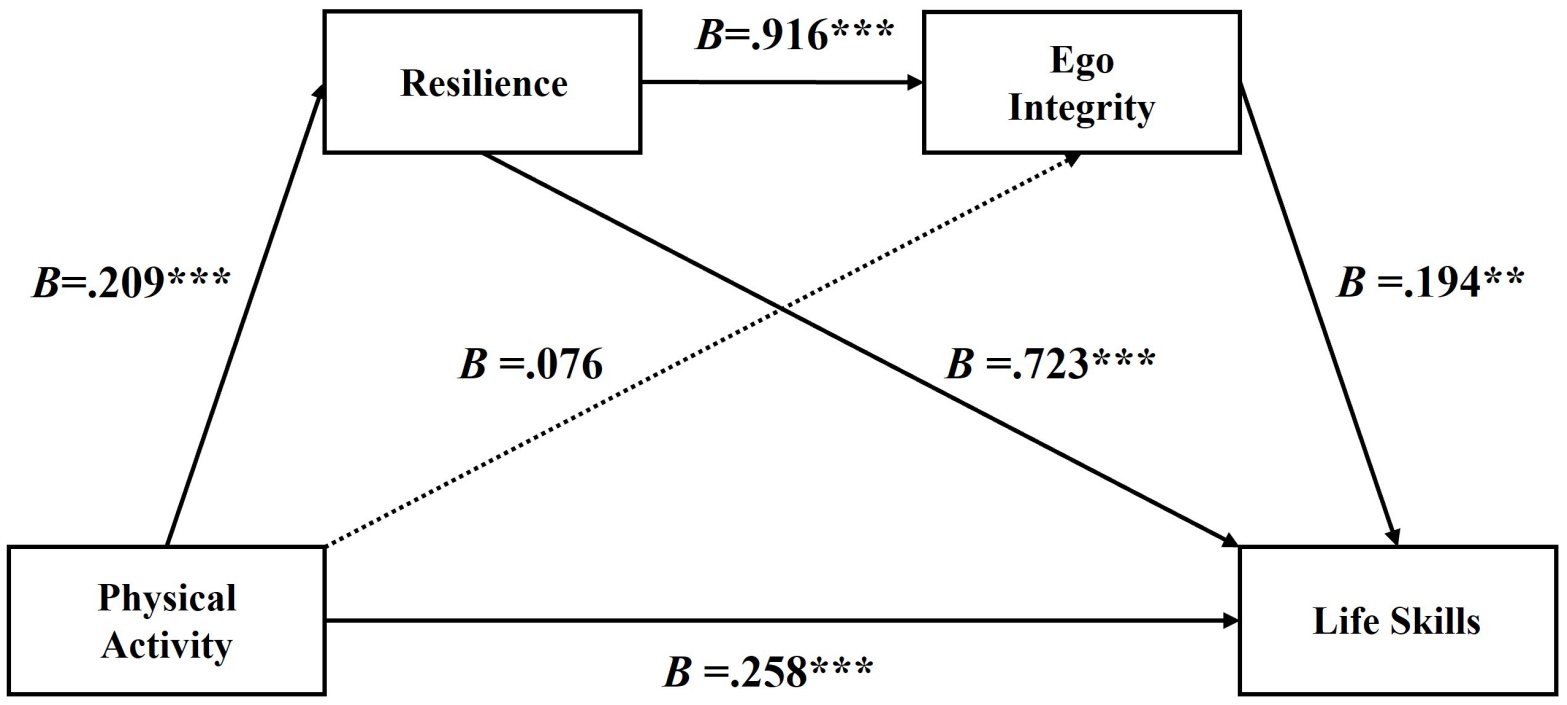
Results of the sequential mediation model. Values on the paths represent regression coefficients (B). The dashed line indicates a non-significant path (*p* > 0.05). ** *p* < 0.01, ***p < 0.001.

The hypothesized indirect association patterns were examined using Hayes’ PROCESS Macro (Model 6). As illustrated in Figure 2, the model explained significant variance in the endogenous variables. Specifically, Physical Activity (PA) participation was significantly and positively associated with resilience (*B* = 0.209, *p* < 0.001, *R^2^* = 0.05). In the subsequent step, resilience showed a significant relationship with ego integrity (*B* = 0.916, *p* < 0.001), explaining a substantial portion of its variance (*R^2^* = 0.54). Notably, the direct path from PA participation to ego integrity was not statistically significant (*B* = 0.076, *p* = 0.123), suggesting that the association between PA and ego integrity may operate indirectly through psychological factors. Finally, in the complete model, PA participation (*B* = 0.258, *p* < 0.001), resilience (*B* = 0.723, *p* < 0.001), and ego integrity (*B* = 0.194, *p* = 0.003) were all positively related to life skills in older adults. The final model accounted for approximately 58% of the total variance in life skills in older adults (*R^2^* = 0.58, *F* = 123.548, *p* < 0.001). The total association between PA participation and life skills was significant (*B* = 0.461, *p* < 0.001), and its direct association remained significant (*B* = 0.258, *p* < 0.001) after accounting for the intermediate variables, suggesting a pattern in which both direct and indirect associations were observed. The detailed results of these path analyses are presented in Table 3.

**Table 3:** Results of the sequential mediation analysis.

Predictors	Outcomes	*B*	*SE*	*t*	*F*	*R^2^*
PA	Resilience	0.209	0.053	3.913***	15.311	0.052
PA	Ego integrity	0.076	0.048	1.587	167.485	0.548
Resilience		0.916	0.053	17.386***		
PA	Life skills	0.258	0.050	5.141***	123.548	0.574
Resilience		0.723	0.079	9.132***		
Ego integrity		0.194	0.062	3.104**		
Total effect		0.461	0.069	6.693***	44.802	0.139

PA = participation in physical activity level (Dummy-coded: 0 = <150 min, 1 = ≥150 min). **p < 0.01, ***p < 0.001.

### 3.3 Indirect effects analysis: sequential mediation

To examine the statistical significance of the indirect pathways within the hypothesized model, bias-corrected bootstrapping with 5,000 resamples was employed. The results indicated that the total indirect association between physical activity (PA) participation and life skills in older adults was statistically significant. Specifically, the pathway involving resilience showed a statistically significant indirect association, as the 95% confidence interval did not include zero (Effect = 0.151, 95% CI [0.072, 0.238]). Conversely, the pathway involving ego integrity alone was not statistically significant (Effect = 0.015, 95% CI [−0.005, 0.044]), suggesting that PA participation may not be related to life skills through ego integrity in isolation. Most importantly, the sequential pathway involving resilience and ego integrity showed a statistically significant indirect association (Effect = 0.037, 95% CI [0.005, 0.083]). These findings suggest that PA participation may be associated with life skills in older adults through statistically significant indirect associations involving resilience and ego integrity. The results of these indirect effects are presented in Table 4. However, the cross-sectional nature of the data limits causal interpretation of these relationships. In addition, because physical activity was operationalized as overall weekly volume rather than by specific activity type or participation context, these results should not be interpreted as indicating that the observed associations were attributable solely to physical activity per se.

**Table 4:** Bootstrapping results for indirect effects.

Predictor	Mediation path	Outcome	Effect	SE	95% CI
PA	→ Resilience →	Life skills	0.151	0.043	[0.072, 0.238]
PA	→ Ego integrity →	Life skills	0.015	0.012	[−0.005, 0.044]
PA	→ Resilience → Ego integrity →	Life skills	0.037	0.020	[0.005, 0.083]
	Total indirect effect		0.203	0.051	[0.103, 0.303]

PA = Physical Activity; CI = Confidence Interval; Bootstrap sample size = 5,000.

## 4 Discussion

### 4.1 Participation in physical activity and life skills in older adults

As hypothesized (H1), the findings of this study indicate that participation in physical activity was significantly and positively associated with life skills in older adults. According to the World Health Organization (15), older adults who meet the recommended threshold of at least 150 min of moderate-intensity physical activity per week tend to demonstrate better physical and mental health, functional ability, and quality of life. In line with this, the present study found that individuals engaging in physical activity at or above the recommended level reported higher levels of life skills in older adults across multiple domains, including physical activity performance skills, daily self-efficacy skills, adaptation and acceptance skills, participation skills in social activities, and engagement skills in digital society (5).

These findings are consistent with previous research indicating a relationship between sport participation and the development of transferable life skills (20, 21, 24). Skills acquired in physical activity settings such as self-regulation, communication, and leadership have been shown to extend to broader life domains. The present study suggests that a similar pattern may be observed in older adulthood, with outcomes reflected in life skills in older adults (24).

In particular, regular participation in physical activity may provide a structured context in which older adults repeatedly practice and reinforce multidimensional competencies. These include physical activity performance skills, daily self-efficacy skills, adaptation and acceptance skills, participation skills in social activities, and engagement skills in digital society, all of which support functional independence and social integration (5, 50). Despite these advantages, most life skills research has focused on Positive Youth Development (PYD), with limited attention given to older populations. Therefore, this study broadens the scope of life skills research by highlighting its relevance in later life.

These findings further suggest that the relationship between physical activity and life skills in older adults may not simply reflect a linear pattern in which “more is always better,” but rather indicate that meeting the recommended threshold of physical activity may function as an important criterion. This interpretation supports the notion that public health guidelines for physical activity represent not merely policy recommendations, but empirically supported benchmarks associated with health and psychological outcomes in later life.

Importantly, the physical activity variable in the present study should be interpreted as an indicator of overall health-promoting physical activity participation rather than as a measure of a specific exercise modality or standardized intervention. This approach is consistent with prior research that has treated physical activity as a broad behavioral construct encompassing diverse domains and contexts. For example, dose–response meta-analytic evidence has synthesized physical activity exposure across studies with diverse assessment methods and exposure definitions, focusing on overall activity volume rather than a single exercise type (17). In addition, a Lancet review of physical activity correlates emphasized that physical activity is shaped by individual, social, environmental, and contextual factors, including self-efficacy and motivation (51). Similarly, a systematic review of older adults examined the association between social support and physical activity across multiple physical activity domains, including leisure-time, transport, household, and occupational activity (52). Therefore, the associations observed in the present study may reflect not only bodily movement or energy expenditure, but also broader participation-related features, such as regular engagement, structured routines, perceived competence, self-regulation, and social interaction. Accordingly, the findings should be understood as associations with overall self-selected health-promoting physical activity participation, rather than as evidence that a specific type of physical activity or physical activity itself alone produced the observed psychological differences.

### 4.2 The role of resilience in the relationship between participation in physical activity and life skills in older adults

A key finding of this study is the role of resilience in the relationship between participation in physical activity and life skills in older adults (H2). Resilience is understood as a dynamic psychological resource that develops through interactions between individuals and their environments (28). In older adulthood, it represents a critical capacity for adapting to stressors such as chronic illness and functional decline. Previous studies have reported positive relationships between physical activity and resilience, with even short-term interventions being linked to improvements in resilience (36). A study by Yang, Zhang, and Li (53) reported that mind–body exercise was associated with improved quality of life through the sequential roles of perceived social support and psychological resilience, thereby suggesting a pathway of “physical activity → psychological protective factors → quality of life,” which supports a mechanism similar to that observed in the present study.

The findings of this study indicate that higher levels of older adults’ participation in physical activity were related to higher levels of resilience, which in turn were associated with enhanced life skills. This pattern suggests that engaging in regular physical activity may provide repeated opportunities to manage and overcome everyday challenges. Through these experiences, individuals may develop resilience-related capacities such as persistence, adaptive coping, and positive reinterpretation, which may subsequently contribute to broader life skills in older adults across multiple domains. Taken together, these findings imply that resilience may represent an important psychological resource underlying the association between physical activity and life skills in older adults (24, 36).

Although physical activity was significantly associated with resilience, it accounted for only a small proportion of the variance in resilience (*R*^2^ = 0.052). This finding is consistent with previous research suggesting that resilience in community-dwelling older adults is a multidimensional construct influenced by multiple psychological, social, health-related, and environmental factors. A systematic review and correlational meta-analysis by Górska et al. (30) found that resilience was associated with a broad range of personal and contextual factors, with individual predictors generally demonstrating small-to-moderate associations. Likewise, Windle (28) conceptualized resilience as a dynamic process of negotiating, adapting to, and managing adversity through resources within the individual, life context, and broader environment, while Southwick et al. (27) emphasized that resilience develops through the interaction of biological, psychological, social, and environmental factors. Therefore, the relatively low explanatory power observed in the present study does not diminish the importance of physical activity. Rather, it suggests that physical activity should be considered one meaningful behavioral resource within a broader constellation of protective factors that jointly support resilience in later life. Future studies should incorporate additional psychological, social, health-related, and environmental variables to more comprehensively explain resilience and its contribution to life skills among older adults.

### 4.3 Sequential roles of resilience and ego integrity in the relationship between participation in physical activity and life skills in older adults

One of the main contributions of this study is the identification of a sequential association involving resilience and ego integrity (H4). The findings indicated that older adults’ participation in physical activity was associated with life skills through a pathway involving resilience and ego integrity, whereas ego integrity alone did not show a significant role when examined independently.

This pattern suggests that improvements in physical functioning and activity levels may not directly correspond to ego integrity, which represents a higher-order developmental process. According to Erikson’s theory, ego integrity involves reflecting on one’s life and integrating both positive and negative experiences into a coherent whole (37, 38, 41, 54). As such, because ego integrity is conceptualized as a complex developmental task involving the acceptance and integration of one’s entire life, it may not be directly achieved through older adults’ participation in physical activity alone. Rather, it is likely to require accumulated psychological experiences and internal adaptive resources that develop over time.

In this context, the present findings suggest that resilience may function as an important psychological resource underlying this process. Previous studies have highlighted that ego integrity is closely related to psychological adaptation, meaning-making, and social relationships (55). Consistent with this perspective, resilience in the present study was positively related to ego integrity, which in turn was associated with life skills in older adults, suggesting that ego integrity may emerge on the basis of adaptive psychological resources.

Furthermore, these findings imply that the experiential benefits of physical activity may not be sufficient, on their own, to support the development of ego integrity. Instead, such experiences may be reflected in resilience, which may subsequently support individuals in reinterpreting life events and engaging in acceptance-oriented processes. This interpretation aligns with the framework proposed by Windle (28), which conceptualizes resilience as a dynamic process of adaptation that supports psychological functioning in later life.

Taken together, the results suggest a sequential pattern in which participation in physical activity may be linked to resilience, which may in turn be associated with processes related to ego integrity, and ultimately be reflected in life skills in older adults (28, 55). This perspective provides a more nuanced understanding of how behavioral engagement may be connected to higher-order developmental outcomes in older adulthood.

### 4.4 Practical and theoretical implications

Theoretically, this study fills a gap in the gerontological literature by integrating observable physical behavior with internal maturation processes. It demonstrates that life skills in later life are not static traits but dynamic capacities that may be associated with psychological processes involving resilience and ego integrity. By highlighting the sequential role of resilience, this research extends Kwon et al. (24), who reported that older adults’ participation in physical activity was associated with life skills and successful aging. Specifically, the findings outline a pathway linking behavior (physical activity), psychological resources (resilience), existential integration (ego integrity), and functional competencies (life skills in older adults). This perspective extends the concept of life skills from youth development to successful aging.

From a practical perspective, the findings suggest that physical activity programs for older adults should not only aim to increase activity levels but also intentionally incorporate elements that support resilience and ego integrity. This may include structured challenges, opportunities for repeated attempts, feedback, and group-based activities that foster social interaction.

In addition, integrating reflective components such as life review, storytelling, and intergenerational exchange may help connect physical activity experiences with the development of ego integrity. At a broader level, integrated programs combining physical activity, psychological support, and digital education may provide a comprehensive approach to enhancing life skills in older adults.

### 4.5 Limitations and future directions

Despite its theoretical and practical contributions, this study has several limitations that suggest directions for future research.

First, due to the cross-sectional design and reliance on self-reported data, causal relationships cannot be inferred. Future research should employ longitudinal or intervention designs to better understand potential pathways among participation in physical activity, resilience, ego integrity, and life skills in older adults. In addition, although Harman’s single-factor test suggested that common method bias was unlikely to be a serious concern, the possibility of common method bias cannot be fully excluded because the main variables were assessed using self-report questionnaires collected at a single time point. Furthermore, although interviewer-assisted administration was provided only for participants with visual or physical limitations, and the researchers were instructed to read each question verbatim without providing additional explanations or suggestions, this approach may have introduced some degree of social desirability bias compared with self-administered questionnaires. Therefore, the findings should be interpreted with this potential source of bias in mind.

Second, because participants were recruited from senior universities and community welfare centers, the sample may overrepresent older adults who were relatively socially engaged, health-conscious, and physically or cognitively capable of participating in community-based activities. Future studies should aim for a more representative and diverse sample to enhance the external validity of the results.

Third, although this study focused on resilience and ego integrity as key psychological mechanisms, other potential variables such as social support, cognitive function, or digital literacy may also play significant roles in the development of life skills in older adults. In particular, given the increasing importance of digital participation in later life, investigating the interplay between physical engagement and digital life skills remains a promising area for future inquiry.

Finally, while the WHO 150-min threshold provided a clear and clinically meaningful benchmark for physical activity, the present study did not examine the specific types, intensities, settings, or contextual and social characteristics of activities. For example, the study did not distinguish between individual and group-based activities, structured exercise programs and self-selected activities, or aerobic and resistance-based activities. In addition, because physical activity was operationalized using a threshold-based categorical approach, some variability in activity levels may not have been fully captured. Future research should therefore investigate how different modalities, social contexts, and continuous levels of physical activity are associated with psychological maturation in order to provide more tailored intervention strategies for the aging population.

## 5 Conclusion

The present study provides empirical evidence regarding the structural relationships linking physical activity and life skills in older adults. By identifying a sequential association involving resilience and ego integrity, this research extends beyond a sole focus on physiological health and highlights the psychological processes associated with successful aging.

The findings suggest that meeting the World Health Organization (WHO) physical activity guidelines may be associated with higher levels of psychological resilience, which may, in turn, be related to ego integrity, a key developmental task in later life. This pattern may ultimately be reflected in multidimensional life skills in older adults.

Given the rapid global aging trend, these results imply that integrated approaches combining physical activity with psychological support may be beneficial for promoting adaptive capacities in older adults. This study contributes to the development of a conceptual framework for future geriatric research and offers practical insights for designing more comprehensive health promotion programs.

## Data Availability

The raw data supporting the conclusions of this article will be made available by the authors, without undue reservation.
